# Myricitrin Inhibits Acrylamide-Mediated Cytotoxicity in Human Caco-2 Cells by Preventing Oxidative Stress

**DOI:** 10.1155/2013/724183

**Published:** 2013-10-09

**Authors:** Wei Chen, Lina Feng, Yang Shen, Hongming Su, Ya Li, Jingjing Zhuang, Lingxia Zhang, Xiaodong Zheng

**Affiliations:** ^1^Department of Food Science and Nutrition, Fuli Institute of Food Science, Zhejiang Key laboratory for Agro-Food Processing, Zhejiang University, Hangzhou 310058, China; ^2^College of Food Science and Biotechnology, Zhejiang Gongshang University, Hangzhou 310035, China

## Abstract

Oxidative stress was thought to be associated with acrylamide cytotoxicity, but the link between oxidative stress and acrylamide cytotoxicity in the gastrointestinal tract, the primary organ in contact with dietary acrylamide, is still unclear. This study was conducted to evaluate the antioxidant activity of natural dietary compound myricitrin and its protective role against acrylamide cytotoxicity. We found that myricitrin can effectively scavenge multiple free radicals (including DPPH free radical, hydroxyl radical, and ABTS free radical) in a concentration-dependent manner. Our results further indicated that the presence of myricitrin (2.5–10 **μ**g/mL) was found to significantly inhibit acrylamide-induced cytotoxicity in human gastrointestinal Caco-2 cells. Moreover, acrylamide-induced cytotoxicity is closely related to oxidative stress in Caco-2 cells. Interestingly, myricitrin was able to suppress acrylamide toxicity by inhibiting ROS generation. Taken together, these results demonstrate that myricitrin had a profound antioxidant effect and can protect against acrylamide-mediated cytotoxicity.

## 1. Introduction

Acrylamide, a white odorless crystalline solid, has been identified in heat-treated carbohydrate-rich foods such as fried cookies, potatoes, bread, and breakfast cereals [1]. Research observations revealed that acrylamide levels appeared to rise as food is subjected to heat for longer periods of time, which might be produced by the reaction between asparagine and reducing sugars (fructose, glucose, etc.) or reactive carbonyls at temperatures above 120°C [2]. Many scientists proposed that the Maillard reaction [2, 3], which was a vital factor to produce brown color and specific taste of bakery food, was responsible for the formation of acrylamide. However, this substance originated toxic effects on nervous system and on fertility [4]. In addition, acrylamide may induce carcinogenesis and heritable mutations in rats when orally administrated in high-dose experiments in laboratory, which implies that it is probably carcinogenic to humans [5, 6]. Consequently, the potential harmfulness of acrylamide cannot be ignored.

In recent years, some studies have demonstrated that acrylamide-induced cytotoxicity was relevant to oxidative stress [7, 8]. The cytotoxic properties of acrylamide by affecting the cellular redox status might lead to generation of reactive oxygen speices (ROS), which would ultimately cause cytotoxic and genotoxic effects. Considering this, researchers endeavor to delve some strategies to reduce acrylamide-mediated cytotoxicity [8–10]. Among dietary compounds, nutritional ingredients extracted from conventional herbal plants attracted a great deal of attention. For instance, Cao et al. reported that curcumin could attenuate acrylamide-induced cytotoxicity and genotoxicity in HepG2 cells by ROS scavenging [8].

Myricitrin (3′, 4′, 5′, 5, 7-five hydroxyflavone-3-O-*α*-L-rhamnoside) is a naturally occurring flavonoid derived from Chinese bayberry bark and fruit as well as other medicinal plants [11, 12]. Its polyhydroxy structure may contribute to potent antioxidative and free radical scavenging activities. Some studies have shown other beneficial properties of myricitrin besides its antioxidative properties [13], such as antiviral and antimicrobial activities [14]. Moreover, this flavonoid possesses anticarcinogenic [15, 16] and antinociceptive [17] activities *in vitro*. Although studies have documented that myricitrin can reduce oxidative stress and cytotoxicity in some tissues and cell lines [18], little information was available with respect to myricitrin as an antioxidant to attenuate acrylamide-mediated cytotoxicity. Furthermore, considering that acrylamide is readily absorbed through gastrointestinal tract, in this study, human gastrointestinal Caco-2 cells were used as an experimental model. Therefore, the aim of this study was to determine the effect of myricitrin to reduce acrylamide-mediated cytotoxicity of Caco-2 cells in order to uncover the mechanism underlying the action of this flavonoid to reduce cytotoxicity.

## 2. Material and Methods

### 2.1. Reagents

Myricitrin (purity > 98%) was obtained from the Chinese National Institute for the Control of Pharmaceutical and Biological Products (Beijing, China). Acrylamide, 6-hydroxy-2,5,7,8-tetramethylchromane-2-carboxylic acid (Trolox), gallic acid, 2,2′-azino-bis(3-ethylbenzothiazoline-6-sulfonic acid) diammonium salt (ABTS), 2,2-diphenyl-1-picrylhydrazyl (DPPH), 2,4,6-tris(2-pyridyl)-1,3,5-triazine (TPTZ), dichlorodihydrofluorescein diacetate (DCFH-DA), and Folin & Ciocalteu's phenol reagent were purchased from Sigma Chemical (St. Louis, MO, USA). All other reagents used were of analytical grade.

### 2.2. DPPH Free Radical Scavenging Assay

The scavenging effects of the samples on the DPPH radical were monitored according to the previous report [19] with slight modification. Briefly, 100 *μ*L of the extract solution was added to 3.9 mL of DPPH solution (0.1 mM), vortexed, and then left to stand at room temperature for 30 min in the dark. The absorbance was measured at 517 nm and activity was expressed as percentage DPPH scavenging relative to control using the following equation:

(1) DPPH  radical-scavenging  activity  (%) =[(Acontrol−Asample)Acontrol]×100.

### 2.3. ABTS Free Radical Scavenging Assay

The ABTS assay was determined according to the previous report [20] with slight modification. ABTS^∙+^ stock solution was produced by reacting 10 mL of 7 mM ABTS solution with 179 mL of 140 mM potassium persulfate aqueous in the dark at room temperature for 12 h before use. When used for analysis, the ABTS^∙+^ stock solution was diluted 20-folds with phosphate-buffered saline (5 mmol/L, pH 7.4). 50 *μ*L of the diluted samples was added to 50 *μ*L of 80% methanol. The ABTS^∙+^ cation solution (3.9 mL) was then added and mixed thoroughly. The reaction mixture was kept at room temperature for 10 min in the dark, and the absorbance was recorded at 734 nm. The activity was expressed as percentage ABTS^∙+^ scavenging relative to control using the following equation:

(2) ABTS∙+  scavenging  activity  (%) =[(Acontrol−Asample)Acontrol]×100.

### 2.4. Ferric Reducing Antioxidant Power (FRAP) Assay

The ferric reducing ability of samples was determined according to the previous study [21]. To prepare the FRAP reagent, a mixture of 0.1 M acetate buffer (pH 3.6), 10 mM TPTZ, and 20 mM ferric chloride (10 : 1 : 1, v/v/v) was made. The FRAP reagent (3.9 mL) was added to 0.1 mL sample solution and mixed. The absorbance was recorded at 593 nm, and the reaction was monitored for 10 min. Trolox standard solution was used to perform the calibration curves. The ferric reducing ability of samples was expressed as Trolox equivalent antioxidant capacity (TEAC) in milligrams per milligram of dry weight.

### 2.5. Inhibition of Fenton's Reaction

The ability of myricitrin to scavenging ^∙^OH was carried out according to the previous report [22]. Briefly, appropriate dilution of samples were added to a reaction mixture containing 60 *μ*L 5 mM 1,10-phenanthroline monohydrate, 560 *μ*L 0.2 M phosphate buffer, 20 *μ*L 0.001% hydrogen peroxide, and 60 *μ*L 5 mM FeSO_4_. The reaction mixture was incubated at 37°C for 30 min. The absorbance was measured at 536 nm. The percentage (%) ^∙^OH radical scavenging ability was subsequently calculated.

### 2.6. Cell Culture

Human Caco-2 cells were obtained from the Cell Bank of Type Culture Collection of Chinese Academy of Sciences (CBTCCCAS, Shanghai, China). Caco-2 cells were cultured in RPMI 1640 medium (Gibco) containing 10% fetal bovine serum (Gibco), 100 units/mL penicillin, and 100 units/mL streptomycin in a humidified cell incubator with an atmosphere of 5% CO_2_ at 37°C.

### 2.7. Cell Viability Assay

Cell viability was examined by the MTT method as previously described [23, 24]. Briefly, cells were seeded into 96-well microtiter plates at a density of 5 × 10^3^ cells/well. After 24 h of incubation, cells were treated with acrylamide in the presence or absence of myricitrin. After further incubated for 48 h, cells were incubation with MTT (0.5 mg/mL) for 4 h. The formazan precipitate was dissolved in 150 *μ*L DMSO, and the absorbance was detected at 490 nm using a Tecan Infinite M200 microplate reader. Each test was performed in triplicate experiments.

### 2.8. Cellular Reactive Oxygen Species (ROS) Assay

Cellular ROS was examined as previously described with some modifications [25]. Briefly, after treatment, cells were collected and incubated with 10 *μ*M DCFH-DA at 37°C for 30 min. After incubation with the fluorochrome, cells were washed with PBS and immediately analyzed by fluorescence microscope.

### 2.9. Statistical Analysis

All data were expressed as means ± standard deviations (SD) from at least three independent experiments and analyzed by student's *t* test or one-way ANOVA using SPSS (version 16.0). *P* < 0.05 was considered statistically significant. 

## 3. Results and Discussion

### 3.1. DPPH Radical Scavenging Activity

**Figure 1:**
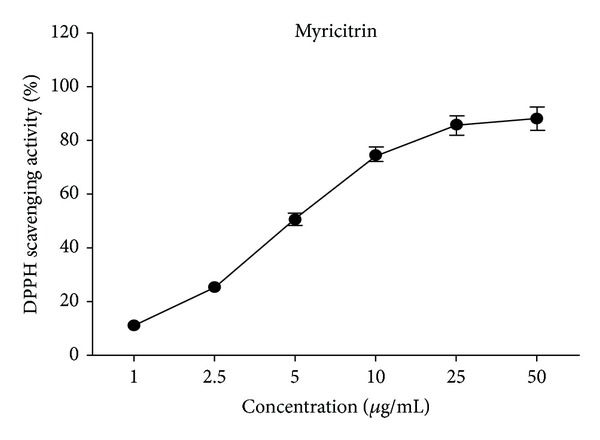
DPPH radical scavenging activity of myricitrin.

DPPH, a stable nitrogen centered free radical, has been widely used to evaluate the free radicals' quenching ability of various natural products and has been accepted as a model compound for free radicals originating in lipids [26]. The bleaching of DPPH absorption (517 nm) by a test compound is representative of its capacity to scavenge free radicals, generated independent of any enzymatic or transition metal-based systems. Hence, the DPPH-scavenging activity of a compound was taken as the parameter to check its antioxidant potential. The results shown in Figure 1 clearly indicate the scavenging activity of myricitrin against DPPH was concentration-dependent (Figure 1). The estimated IC_50_ value of myricitrin (5.64 *μ*g/mL), which stands for the concentration of an antioxidant required to scavenge 50% of the radicals in the reaction mixture, was slightly higher than that of the water-soluble analog of vitamin E, Trolox (2.51 *μ*g/mL) [27], but lower than that of the synthetic antioxidant, BHA (35.5 *μ*g/mL).

### 3.2. ABTS Radical Scavenging Activity

**Figure 2:**
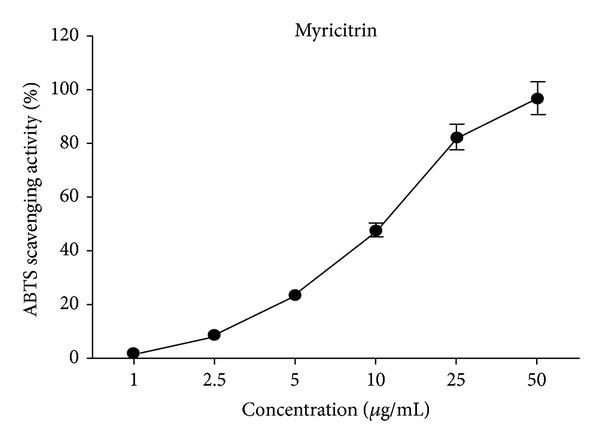
ABTS scavenging activity of myricitrin.

ABTS radical scavenging assay is commonly used in antioxidant studies due to its simplicity and short operation time. ABTS can be converted to its radical cation form in water with the presence of an oxidising factor such as potassium persulfate, 2,20-azo-bis (2-aminopropane), or the binary mixtures of H_2_O_2_ with a peroxidase enzyme [28]. As shown in Figure 2, there was a concentration-dependent bleaching of ABTS^∙+^ by myricitrin. The estimated IC_50_ value of myricitrin for ABTS radical scavenging was 9.67 *μ*g/mL, which was slightly higher than that of the standard Trolox (2.43 *μ*g/mL) [27].

### 3.3. Hydroxyl Radical Scavenging Activity

**Figure 3:**
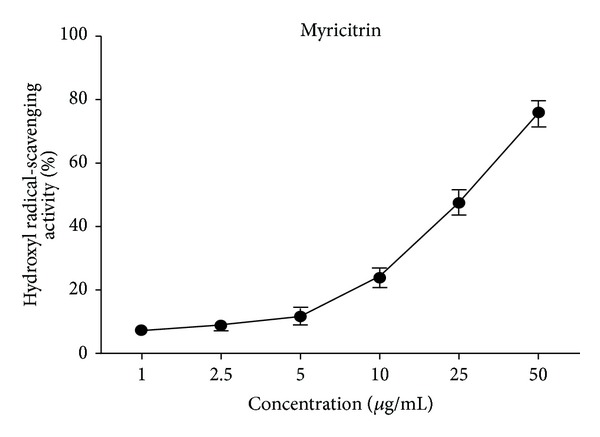
Hydroxyl radical scavenging activity of myricitrin.

Hydroxyl radical, the most reactive free radical species known to date, is harmful to almost every biological molecule found in living cells [29]. Hydroxyl radical can be formed in several ways. Among these, Fenton reaction is the most important mechanism* in vitro,* where a transition metal is involved as a prooxidant in the catalyzed decomposition of superoxide and hydrogen peroxide [30]. In this work, using Fenton reaction, we detected the effect of myricitrin on hydroxyl radical. The results in Figure 3 showed the scavenging activity of myricitrin against hydroxyl radical was concentration-dependent. The estimated IC_50_ value of myricitrin for hydroxyl radical scavenging was 24.81 *μ*g/mL, which was higher than that of the standard Trolox (0.1 *μ*g/mL) [31].

### 3.4. FRAP Assay

The antioxidant activity of myricitrin was also tested by FRAP assay. In this method, the antioxidant activity is determined based on the ability to reduce Fe^3+^ to Fe^2+^ in the presence of TPTZ, forming an intense blue Fe^2+^-TPTZ complex with an absorption maximum at 593 nm [21]. The ferric reducing ability of samples was expressed as Trolox equivalent antioxidant capacity (TEAC) in milligrams per milligram of myricitrin. Similar to the results obtained from the DPPH assay and ABTS assay, myricitrin showed high reducing activity (2.33 mg TEAC)

### 3.5. Effect of Myricitrin on Acrylamide-Mediated Cytotoxicity in Human Caco-2 Cells

**Figure 4:**
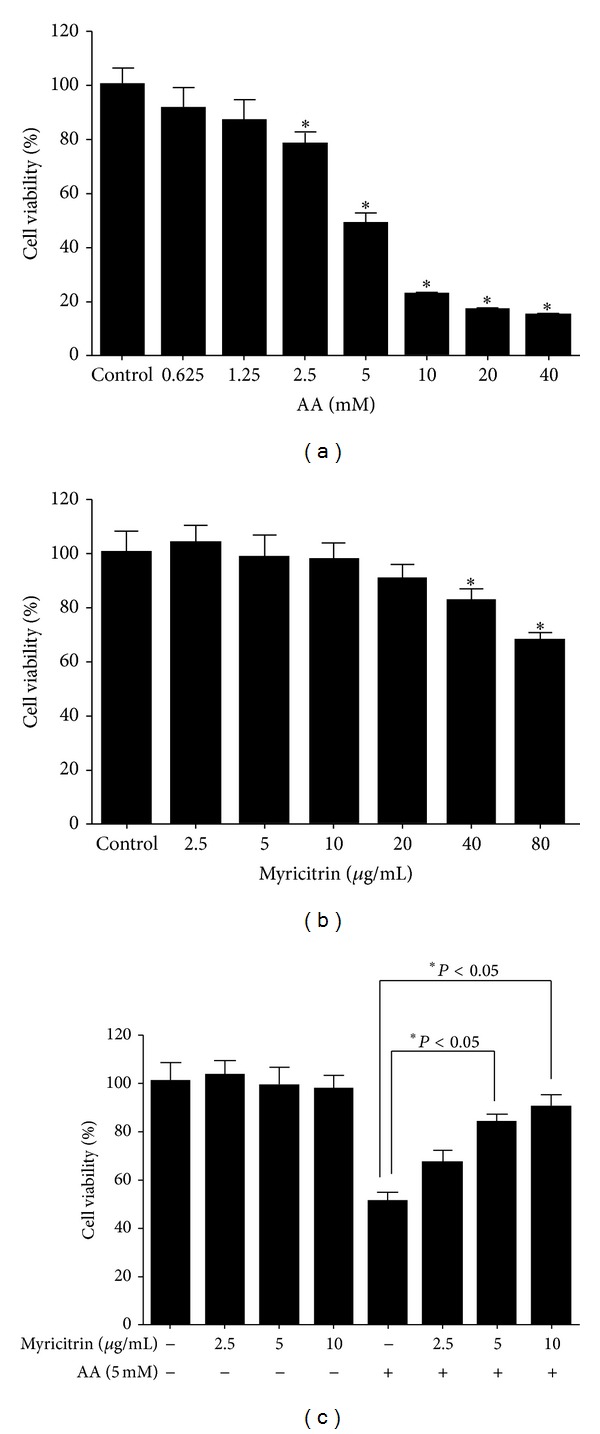
Effect of myricitrin on acrylamide-induced cytotoxity in human Caco-2 cells. The Caco-2 cells were exposed to 5 mM acrylamide for 48 h in the presence or absence of myricitrin; cell viability was detected using MTT method. Data of column represent means ± SD of three independent experiments (*P < 0.05 versus acrylamide treatment group). AA: acrylamide.

Oxidative stress is thought to play an important role in the progression of many chronic diseases including diabetes, cancer, cardiovascular diseases, and neurodegenerative disorders [32–35]. One such mediator of oxidative stress is acrylamide, which could induce neurotoxic, genotoxic, and carcinogenic effects [36]. The highly free radical scavenging activity of myricitrin prompted us to investigate its protective role against acrylamide-induced cytotoxity. In this study, we used human Caco-2 cells to determine the effect of myricitrin on acrylamide-induced cytotoxity. In order to identify the doses at which cell toxicity was induced by acrylamide, Caco-2 cells were exposed during 48 h to different concentrations of acrylamide (0–40 mM). As shown in Figure 4(a), cell viability, as determined by MTT assay, was significantly decreased in the presence of 2.5–40 mM acrylamide. We next examined the ability of myricitrin to protect against acrylamide-induced cytotoxicity. As myricitrin did not decrease cell viability at lower concentrations (<20 *μ*g/mL) (Figure 4(b)), Caco-2 cells were exposed to acrylamide (5 mM) for 48 h in the presence of myricitrin (2.5–10 *μ*g/mL), followed by MTT assay. As illustrated in Figure 4(c), acrylamide-induced cytotoxicity could be inhibited by myricitrin in a concentration-dependent manner. A significant inhibition was detected as little as 5 *μ*g/mL myricitrin. Metabolism study previously revealed that myricitrin was detectable in urine after oral administration of myricitrin (100 mg), which was 1.46 mg/24 h per rat at Day 1 and 0.47 mg/24 h per rat at Day 2, respectively [37], whereas faecal extracts did not detect this substance, suggesting that myricitrin was completely assimilated by the gastrointestinal system, and the concentrations of the myricitrin used in this study could be obtainable under *in vivo* condition. 

### 3.6. Myricitrin Attenuates Acrylamide-Induced Cellular ROS Increase

**Figure 5:**
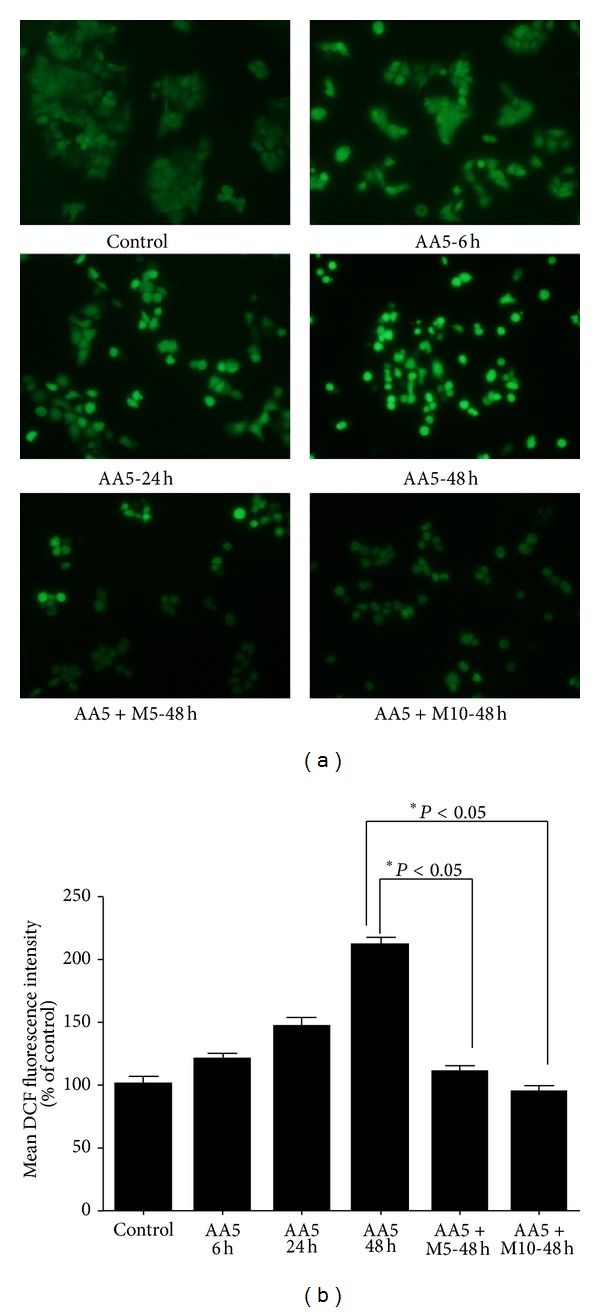
Effect of water extract of bayberry on ROS production in acrylamide-treated Caco-2 cells. (a) After treatment with 5 mM acrylamide in the presence or absence of myricitrin for 0–48 h, Caco-2 cells were incubated with 10 *μ*M DCFH-DA for 30 min and then immediately subjected to fluorescence microscope analysis. (b) The quantitative data of panel (a) and results were expressed as mean DCF fluorescence intensity (means ± SD of three independent experiments). *P value represents significant difference between conditions, where *P* < 0.05. AA: acrylamide. AA5: 5 mM acrylamide; M5: 5 *μ*g/mL myricitrin; M10: 10 *μ*g/mL myricitrin.

The cytotoxic effect of acrylamide is associated with free radicals generation in multiple types of cells [7, 8]. Therefore, we analyzed the production of intracellular ROS in acrylamide-treated Caco-2 cells by fluorescence microscope using DCFH-DA. As shown in Figure 5(a), compared to the untreated cells (control group), acrylamide caused a dramatic increase of ROS generation in Caco-2 cells in a time-dependent manner. The mean DCF fluorescence of cells was 211.1% of the control group after treatment with 5 mM acrylamide for 48 h. This ROS increase could be largely suppressed by pretreatment with 5 *μ*g/mL myricitrin, and further suppression can be observed at 10 *μ*g/mL myricitrin pretreated-group. Together, these results suggest that the myricitrin can effectively attenuate acrylamide-induced ROS increase.

## 4. Conclusions 

In this work, we found that myricitrin had a profound antioxidant effect and inhibited acrylamide-mediated oxidative stress and cytotoxicity in Caco-2 cells. To the best of our knowledge, myricitrin was approved as General Regarded As Safe (GRAS) by Flavour Extract Manufacturers' Association (FEMA), which has been added to food, cosmetic, and pharmaceutical products [38]. Taken together, our results indicate that myricitrin can be used as a natural antioxidant for protection against acrylamide-induced cytotoxicity by preventing oxidative stress.
